# The assessment of phosphorus recovery potential in sewage sludge incineration ashes — a case study

**DOI:** 10.1007/s11356-022-22618-4

**Published:** 2022-09-20

**Authors:** Monika Kasina

**Affiliations:** Institute of Geological Sciences, Jagiellonian University, Gronostajowa 3a, 30-387 Krakow, Poland

**Keywords:** Sequential extraction, Golterman procedure, Incineration sewage sludge ash, Phosphorus recovery

## Abstract

A sewage sludge incineration ash contains large amounts of phosphorus, which are considered as a novel anthropogenic waste–based substitute for phosphorus natural resources. Phosphorus is accumulated at most in phosphate minerals of whitlockite structure, that contain Fe, Ca, and Mg and in the matrix composed of Si, Al, Fe, Ca, P, Mg, K, Na in various proportions. The goal of this study was to estimate phosphorus recovery potential. A four-stage sequential extraction, following the modified Golterman procedure, was applied. Separation of four independent fractions enabled to understand better the manner of phosphorus occurrence in the studied ash. The results of the extraction indicated the greatest release of phosphorus combined with organic matter using sulfuric acid. The release was on average at the level of 64%. The chelating Na-EDTA compound indicated lower ability to extract phosphorus (at the level of 35%), and the highest ability to extract heavy metals and potentially toxic elements (As, Zn, Mo). The sequential extraction led to the total recovery of phosphorus of around 40–60%

## Introduction

Phosphorus macronutrient is a key element for the growth of life; however, geological non-renewable in human scale phosphorus resources are plainly limited (Alewell et al. 2020; Meng et al. 2021). It is estimated that phosphorus reserves will be exhausted within up to 400 years depending on demands and use (Van Dijk et al. 2016). For these reasons, phosphorous was recognized by European Commission as a critical raw material (European Commission 2014). This is related to the fact that the demand for phosphorus increases significantly. European Union countries do not have their own phosphate deposits that extraction would be profitable. The main reserves of phosphate rocks and global raw material production is focused around China, USA, and Morocco and Western Sahara (Gorazda et al. 2013). On the other hand, the low efficiency in phosphorus cycle causes phosphorus excess in nature. Due to losses related to phosphorus mining, processing, phosphate fertilizer production, and usage, serious environmental pollution occurs including not only eutrophication which influences water quality and biodiversity (Steffen et al. 2015), but also loss on landscape quality, greenhouse gas emissions, and fresh water consumption (Schroder et al. 2009). Additionally, vast amounts of phosphorus end up in wastewater (Cordell et al. 2009). A stable supply for phosphorus raw materials and management is one of the major challenges for economies nowadays.

Approximately 90% of the incoming phosphorus load is present in the sewage sludge (Desmidt et al. 2015). In the liquid phase, it can reach 40–50%, while recovery rates from sewage sludge and sewage sludge ash can reach up to 90% (Cornel and Schaum 2009).

The most common option to recover phosphorus was its extraction from wastewater by, e.g., by struvite precipitation (NH_4_MgPO_4_ 6H_2_O) (e.g., Jaffer et al. 2002; Shu et al. 2006; Le Corre et al. 2009); however, this method is expensive due to high cost of sterilization. The simplest and cheapest method is the direct application of sewage or sewage sludge as fertilizer, but it is significantly limited by EU law due to the presence of toxic elements in the sewage sludge, harmful organic substances, bacteria, and parasites (e.g., Belhaj et al. 2016; Lamastra et al. 2018). Since, sewage sludge contains not only large amounts of phosphorus but also other nutrients such as K, Na, Ca, and Mg, it may be directly used by plants in dump site reclamation process (Antonkiewicz 2010; Antonkiewicz et al. 2018).

Another interesting option where high phosphorus recovery rates are obtained is thermochemical treatment with chlorine-based additives, where the phosphorus fixation rate reaches 98.5% (Yang et al. 2019).

A potential source of phosphorus is sewage sludge incineration ash. Waste stream materials such as sewage sludge incineration ashes are more often considered as novel anthropogenic waste–based substitutes for natural resources (Kasina et al. 2020), thus maximizing natural resources protection and returning elements into the production cycle by recycling, reuse and at the same time fulfilling assumptions of close-loop economy. Obtaining phosphorus from a sewage sludge incineration ashes seems to be a reasonable and promising option, since the average phosphorus content corresponds to medium-rich ores (Kasina et al. 2021). Another important aspect in favor of using sewage sludge incineration ash as a phosphorus source is the fact that their amount of fly ash will increase in the coming years, since waste incinerations is considered as one of the most reasonable options to significantly reduce the amount of landfilled waste. Additionally the easy availability of material and the expected increase in exploitation prices of natural deposits will cause fly ash an extremely attractive and cost-competitive material in terms of phosphorus recovery.

The goals of this study were:

- to evaluate the raw material potential of sewage sludge incineration ashes from one of the Polish incineration plants located in the South of Poland;
- to identify and quantify a share of various forms of phosphorus
- to characterize phosphorus mineral phases

To achieve the goals, a four-stage sequential extraction based on the modified Golterman procedure (Golterman 1996) was applied.

Over the last years, numerous works have been published on the raw material potential of incineration ashes, and different recovery methods have been tested. Nevertheless, due to the fact that ashes from different locations differ from each other, it is necessary to approach individually the incineration residues. These differences are related to various aspects such as the level of advancement of local waste management, seasonal changes, the level of development of the region which can strongly influence the composition of ashes, and thus their recovery potential as well as release potential of undesirable components such as environmentally toxic elements.

## Materials and methods

For the purpose of this study, fly ash from sewage sludge incineration plant located in the South of Poland was considered.

Four sewage sludge incineration ash (ISSA) samples, namely SZ01, SZ02, SZ03, and SZ04 were collected from the plant with 3–4 months intervals: autumn, spring, summer, and winter, respectively. Details concerning collection of samples and their affiliation are listed in Table 1.

**Table 1:** ISSA classification and sampling time

	Sample	Waste code	Sampling
ISSA	SZ01	19 01 14^1^	November 2015
	SZ02	19 01 14	March 2016
	SZ03	19 01 14	July 2016
	SZ04	19 01 14	December 2016

^1^Fly ash other than those mentioned in 19 01 13*, where 19 01 13* is a fly ash containing dangerous substances

The ISSA is classified as non-hazardous waste in accordance with the Guidance on classification of waste according to EWC-Stat categories (2010) and Annex III to Commission Decision of 18 December 2014 amending Decision 2000/532/EC on the list of waste pursuant to Directive 2008/98/EC of the European Parliament and of the Council, n. d). It is red in color, fine-grained material composed of quartz, feldspar, hematite, and phosphorus-rich phases (Kasina et al. 2019) as main mineral phases.

### Sampling site

A sewage sludge incineration plant was located in the South of Poland in a ca. 800,000 population city. There, partly dewatered sludge is incinerated in a fluidized bed boiler (supplier Pyrofluid™) at 850–900 °C. Fluidization is obtained by the addition of silica sand and air stream introduction at high pressure. Detailed technical aspects of incineration facility are described in Kasina et al. (2019).

### Sequential extraction

**Fig. 1:**
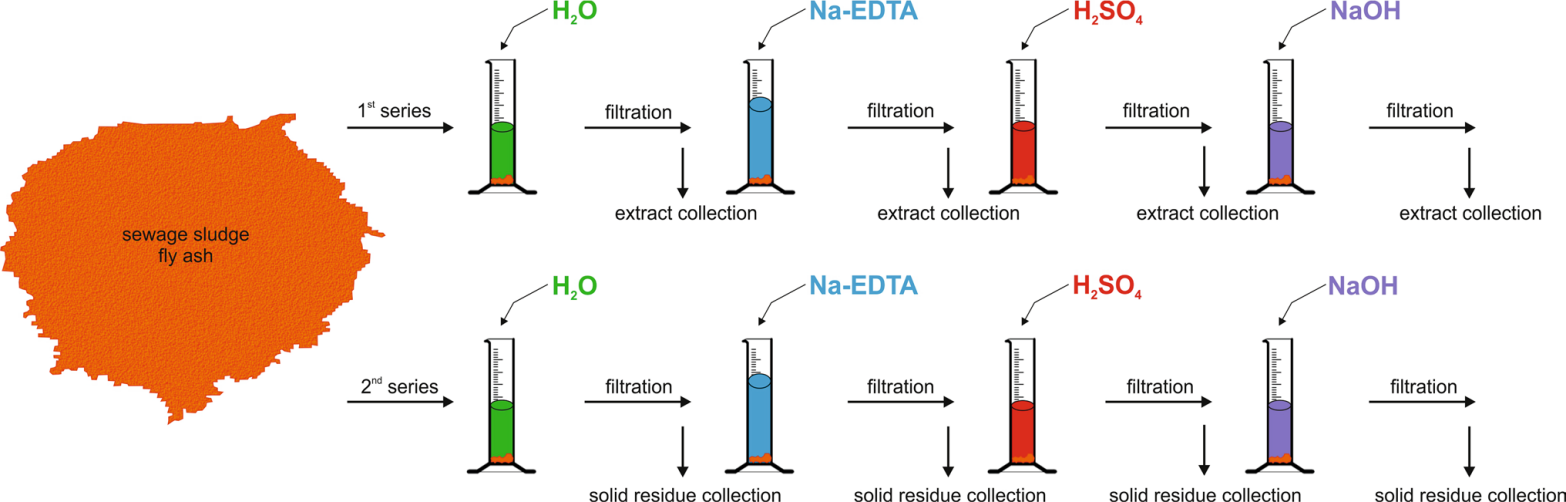
The overview of experimental setup

A four-stage sequential extraction based on the modified Golterman method from 1996 (Fig. 1) was applied, in order to separate four individual fractions (F), using the following chemical reagents:

- F1 — a 24-h extraction with deionized water — allowed for the removal of easily soluble components
- F2 — an 18-h extraction with 0.1 M Na-EDTA — phosphorus bound with carbonates
- F3 — a 2-h extraction with 0.5 M sulfuric acid — phosphorus from soluble compounds with organic matter
- F4 — a 2-h extraction with 2 M NaOH solution — residual phosphorus, bond with aluminosilicates and in bonds that have not been broken in the reaction sulfuric acid.

This procedure was chosen due to the fact that it delivers well-defined fractions and gives an information about bioavailability in the specific fraction (Wang et al. 2013).

In accordance with the assumption of the method, the chemical reagents do not dissolve the extracted phosphorus compounds.

The sequential extraction was carried out in two parallel series which enable verification of results. In a set of four averaged ISSA samples (6 g each; in the original procedure), the extraction was performed on 1 g of the sample, and in order to obtain the appropriate amount of material for mineralogical analyses, the sample mass and the volume of chemical reagents was increased accordingly. After each extraction step, the pH was measured using Elmetron CP-401 pH-meter, which, prior to measurement, was calibrated using standard buffering solutions (pH = 4, pH = 7, and pH = 9) and measured after each step of extraction.

The leachates (100 mL) were poured into polypropylene containers and prepared for analysis. A total of 32 post-extraction solutions (16 from each series) and 1 sample of deionized water as reference material were obtained.

The leachability was calculated using the following formula:

$$R = c\left(\mathrm{solved},\mathrm{ mg }{\mathrm{L}}^{-1}\right) \times V \left(\mathrm{L}\right) \times \left(c{\left(\mathrm{total},\mathrm{ mg}/\mathrm{kg}\right)}^{-1} \times m{\left(\mathrm{sample},\mathrm{ kg}\right)}^{-1}\right)$$

$$R\mathrm{\% }= R \times 100$$

The results of the extraction are listed in Tables 2 and 3.

**Table 2:** The phosphorus concentration in a specific fraction and in total

Sample	*P* % in starting sample	*P* mg/L after leaching	R	R [%]	Share [%]		Sample	*P* % in starting sample	*P* mg/L after leaching	R	R [%]	Share [%]
F1-SZ01-I		0.16	0.00003	0.0034	0.06		F1-SZ01- II		0.21	0.00004	0.004	0.007
F2-SZ01-I	7.81	793	0.17	17.07	34.26		F2-SZ01- II	7.81	801	0.17	17.24	31.15
F3-SZ01-I		1370	0.33	32.53	65.28		F3-SZ01- II		1570	0.37	37.28	67.37
F4-SZ01-I		10-Feb	0.00	0.23	0.46		F4-SZ01- II		36.4	0.01	0.82	1.48
*P* recovery rate				49.83			*P* recovery rate				55.34	
F1-SZ02-I		0.06	0.00	0.001	0.002		F1-SZ02-II		0.06	0.00001	0.001	0.017
F2-SZ02-I	7.74	917	0.20	19.74	32.66		F2-SZ02-II	7.74	914	0.20	19.67	32.56
F3-SZ02-I		1560	0.37	37.04	61.27		F3-SZ02-II		1510	0.36	35.85	59.33
F4-SZ02-I		163	0.04	3.68	44810.00		F4-SZ02-II		217	0.05	4.89	8.09
*P* recovery rate				60.45							60.42	
F1-SZ03-I		0.06	0.00001	0.001	0.003		F1-SZ03-II		0.06	0.00001	0.001	0.002
F2-SZ03-I	7.02	568	0.12	12.23	30.71		F2-SZ03-II	7.02	1210	0.26	26.04	43.37
F3-SZ03-I		1150	0.27	27.30	68.56		F3-SZ03-II		1260	0.30	29.92	53.28
F4-SZ03-I		13	0.003	0.29	0.73		F4-SZ03-II			0.002	0.20	0.36
*P* recovery rate				39.82							56.16	
F1-SZ04-I		0.27	0.0001	0.01	0.02		F1-SZ04-II		0.16	0.00003	0.003	0.005
F2-SZ04-I	7.39	955	0.21	20.56	37.56		F2-SZ04-II	7.39	957	0.21	20.60	36.83
F3-SZ04-I		1240	0.29	29.44	53.78		F3-SZ04-II		1320	0.31	31.34	56.03
F4-SZ04-I		210	0.05	4.74	8.66		F4-SZ04-II		177	0.04	3.99	7.13
*P* recovery rate				54.74							55.93	

*F1*–*F4* fraction, *SZ01*–*SZ04* sample names, *I*, *II* series, *R* leachability, *R*% share of element in the specific fraction in percent

**Table 3:** The concentration of main elements and heavy metals and potentially toxic elements (including REE) in the leachates in a specific fraction based on chemical analyses using ICP methods

	Si			Al			Fe			Mg			Ca			Na			K			Mn			As		
	mg/L after leaching	R	R [%]	mg/L after leaching	R	R [%]	mg/L after leaching	R	R [%]	mg/L after leaching	R	R [%]	mg/L after leaching	R	R [%]	mg/L after leaching	R	R [%]	mg/L after leaching	R	R [%]	mg/L after leaching	R	R [%]	mg/L after leaching	R	R [%]
F1-SZ01-I	1.70	0.00	0.02	1.59	0.00	0.06	0.02	0.00	0.00	66.40	0.05	5.29	162.00	0.03	2.99	18.30	0.06	5.79	43.20	0.05	4.79	0.02	0.00	0.03	0.02	0.02	1.68
F2-SZ01-I	27.50	0.00	0.28	74.10	0.03	2.83	102.00	0.02	1.70	7.35	0.01	0.56	1000.00	0.17	17.33	2010.00	6.10	610.11	39.50	0.04	4.13	12.50	0.24	24.47	0.37	0.44	44.21
F3-SZ01-I	32.30	0.00	0.28	337.00	0.14	14.33	212.00	0.04	4.06	457.00	0.33	33.42	596.00	0.11	11.22	62.90	0.20	19.90	56.90	0.07	6.64	7.11	0.15	15.31	0.06	0.09	9.14
F4-SZ01-I	135.00	0.01	1.24	19.70	0.01	0.73	1.00	0.00	0.02	0.20	0.00	0.01	80.00	0.02	1.88	11500.00	41.00	4100.17	48.80	0.05	5.24	0.01	0.00	0.02	0.02	0.02	2.13
F1-SZ01- II	1.60	0.00	0.02	1.91	0.00	0.08	0.01	0.00	0.00	59.50	0.05	4.74	153.00	0.03	2.82	18.70	0.06	5.92	45.50	0.05	5.05	0.01	0.00	0.02	0.02	0.02	1.66
F2-SZ01- II	27.50	0.00	0.28	74.40	0.03	2.84	86.70	0.01	1.45	6.36	0.00	0.48	940.00	0.16	16.29	2010.00	6.10	610.11	39.70	0.04	4.15	12.70	0.25	24.86	0.39	0.47	46.57
F3-SZ01- II	34.90	0.00	0.31	370.00	0.16	15.73	216.00	0.04	4.13	568.00	0.42	41.53	616.00	0.12	11.60	52.80	0.17	16.70	71.80	0.08	8.38	7.11	0.15	15.31	0.07	0.10	9.99
F4-SZ01- II	198.00	0.02	1.82	20.00	0.01	0.74	1.00	0.00	0.02	0.35	0.00	0.03	133.00	0.03	3.13	11300.00	40.29	4028.87	56.70	0.06	6.09	0.01	0.00	0.02	0.02	0.02	2.37
F1-SZ02-I	1.30	0.00	0.01	3.19	0.00	0.13	0.01	0.00	0.00	43.10	0.03	3.43	144.00	0.03	2.66	20.70	0.07	6.55	36.00	0.04	3.99	0.01	0.00	0.01	0.02	0.02	1.66
F2-SZ02-I	44.30	0.00	0.45	85.90	0.03	3.28	91.50	0.02	1.53	7.90	0.01	0.60	1160.00	0.20	20.10	2010.00	6.10	610.11	45.90	0.05	4.80	12.40	0.24	24.27	0.45	0.53	52.60
F3-SZ02-I	48.20	0.00	0.42	396.00	0.17	16.84	238.00	0.05	4.55	593.00	0.43	43.36	621.00	0.12	11.69	63.70	0.20	20.15	85.10	0.10	9.94	7.62	0.16	16.41	0.08	0.11	10.82
F4-SZ02-I	513.00	0.05	4.71	70.20	0.03	2.58	4.01	0.00	0.07	0.47	0.00	0.03	80.00	0.02	1.88	11600.00	41.36	4135.83	106.00	0.11	11.38	0.01	0.00	0.02	0.02	0.03	2.56
F1-SZ02-II	1.10	0.00	0.01	3.53	0.00	0.14	0.01	0.00	0.00	37.80	0.03	3.01	131.00	0.02	2.42	17.00	0.05	5.38	36.90	0.04	4.09	0.00	0.00	0.01	0.01	0.02	1.50
F2-SZ02-II	43.60	0.00	0.44	83.80	0.03	3.20	89.80	0.02	1.50	7.17	0.01	0.54	1150.00	0.20	19.93	2010.00	6.10	610.11	36.20	0.04	3.79	10.90	0.21	21.34	0.40	0.47	47.04
F3-SZ02-II	47.80	0.00	0.42	387.00	0.16	16.45	221.00	0.04	4.23	565.00	0.41	41.31	619.00	0.12	11.66	60.50	0.19	19.14	91.30	0.11	10.66	5.61	0.12	12.08	0.07	0.10	10.04
F4-SZ02-II	289.00	0.03	2.65	50.00	0.02	1.84	1.00	0.00	0.02	0.96	0.00	0.07	671.00	0.16	15.78	11300.00	40.29	4028.87	61.10	0.07	6.56	0.03	0.00	0.07	0.02	0.03	2.81
F1-SZ03-I	1.60	0.00	0.02	2.87	0.00	0.11	0.02	0.00	0.00	40.70	0.03	3.24	157.00	0.03	2.90	16.20	0.05	5.13	30.50	0.03	3.38	0.00	0.00	0.00	0.01	0.01	1.13
F2-SZ03-I	73.70	0.01	0.74	148.00	0.06	5.66	93.80	0.02	1.57	243.00	0.18	18.35	524.00	0.09	9.08	2000.00	6.07	607.08	54.20	0.06	5.67	1.98	0.04	3.88	0.03	0.04	3.92
F3-SZ03-I	43.40	0.00	0.38	283.00	0.12	12.03	184.00	0.04	3.52	626.00	0.46	45.77	634.00	0.12	11.94	110.00	0.35	34.80	65.00	0.08	7.59	5.46	0.12	11.76	0.07	0.09	9.50
F4-SZ03-I	110.00	0.01	1.01	24.80	0.01	0.91	2.23	0.00	0.04	0.51	0.00	0.04	80.00	0.02	1.88	11500.00	41.00	4100.17	72.70	0.08	7.81	0.02	0.00	0.03	0.00	0.00	0.36
F1-SZ03-II	1.40	0.00	0.01	2.10	0.00	0.08	0.01	0.00	0.00	29.60	0.02	2.36	138.00	0.03	2.55	14.30	0.05	4.52	21.80	0.02	2.42	0.00	0.00	0.00	0.01	0.01	0.90
F2-SZ03-II	47.20	0.00	0.48	309.00	0.12	11.81	199.00	0.03	3.33	687.00	0.52	51.88	625.00	0.11	10.83	89.20	0.27	27.08	72.20	0.08	7.55	5.18	0.10	10.14	0.06	0.07	7.09
F3-SZ03-II	19.50	0.00	0.17	45.20	0.02	1.92	50.00	0.01	0.96	18.70	0.01	1.37	97.00	0.02	1.83	10.70	0.03	3.39	28.60	0.03	3.34	0.76	0.02	1.64	0.02	0.02	2.27
F4-SZ03-II	73.70	0.01	0.68	16.50	0.01	0.61	2.63	0.00	0.04	0.34	0.00	0.02	80.00	0.02	1.88	11300.00	40.29	4028.87	55.30	0.06	5.94	0.03	0.00	0.07	0.00	0.00	0.41
F1-SZ04-I	1.80	0.00	0.02	1.96	0.00	0.08	0.01	0.00	0.00	58.50	0.05	4.66	103.00	0.02	1.90	17.80	0.06	5.63	46.10	0.05	5.11	0.01	0.00	0.01	0.03	0.03	2.84
F2-SZ04-I	29.00	0.00	0.29	97.80	0.04	3.74	99.80	0.02	1.67	10.00	0.01	0.76	1010.00	0.18	17.50	2010.00	6.10	610.11	42.10	0.04	4.40	12.70	0.25	24.86	0.39	0.46	46.34
F3-SZ04-I	35.00	0.00	0.31	461.00	0.20	19.60	190.00	0.04	3.64	506.00	0.37	37.00	655.00	0.12	12.33	410.00	1.30	129.71	76.70	0.09	8.95	5.34	0.11	11.50	0.09	0.13	13.10
F4-SZ04-I	304.00	0.03	2.79	56.30	0.02	2.07	2.84	0.00	0.05	0.80	0.00	0.06	80.00	0.02	1.88	11300.00	40.29	4028.87	82.20	0.09	8.83	0.01	0.00	0.02	0.02	0.02	1.89
F1-SZ04-II	1.80	0.00	0.02	1.56	0.00	0.06	0.01	0.00	0.00	60.40	0.05	4.81	98.70	0.02	1.82	14.50	0.05	4.59	35.40	0.04	3.93	0.01	0.00	0.01	0.02	0.03	2.53
F2-SZ04-II	29.80	0.00	0.30	105.00	0.04	4.01	101.00	0.02	1.69	7.62	0.01	0.58	976.00	0.17	16.91	2010.00	6.10	610.11	36.20	0.04	3.79	11.30	0.22	22.12	0.36	0.43	43.03
F3-SZ04-II	45.00	0.00	0.40	518.00	0.22	22.02	229.00	0.04	4.38	591.00	0.43	43.21	646.00	0.12	12.16	59.40	0.19	18.79	79.90	0.09	9.33	4.63	0.10	9.97	0.07	0.10	9.84
F4-SZ04-II	344.00	0.03	3.16	67.70	0.02	2.49	3.55	0.00	0.06	1.21	0.00	0.09	80.00	0.02	1.88	11300.00	40.29	4028.87	82.10	0.09	8.82	0.02	0.00	0.04	0.02	0.02	2.42

	Cd			Cr			Cu			Mo			Ni			Pb			Zn			∑ REE		
	mg/L after leaching	R	R [%]	mg/L after leaching	R	R [%]	mg/L after leaching	R	R [%]	mg/L after leaching	R	R [%]	mg/L after leaching	R	R [%]	mg/L after leaching	R	R [%]	mg/L after leaching	R	R [%]	mg/L after leaching	R	R [%]
F1-SZ01-I	0.00	0.00	0.02	0.02	0.00	0.03	0.00	0.00	0.00	0.52	0.34	33.79	0.00	0.00	0.01	0.00	0.00	0.00	0.00	0.00	0.00	0.00	0.00	0.00
F2-SZ01-I	0.06	0.20	20.21	0.86	0.02	1.84	5.71	0.14	14.28	0.28	0.22	22.25	0.58	0.11	11.12	1.21	0.16	15.83	38.60	0.16	16.34	0.39	0.06	6.40
F3-SZ01-I	0.07	0.25	24.93	4.15	0.08	8.36	4.74	0.14	13.85	0.04	0.04	3.74	0.26	0.06	5.51	0.15	0.02	2.03	17.50	0.08	8.22	1.00	0.17	17.19
F4-SZ01-I	0.00	0.00	0.29	0.06	0.00	0.19	0.07	0.00	0.17	0.03	0.02	2.12	0.03	0.00	0.49	0.45	0.05	4.99	0.38	0.00	0.16	0.00	0.00	0.02
F1-SZ01- II	0.00	0.00	0.02	0.02	0.00	0.03	0.00	0.00	0.00	0.52	0.34	34.12	0.00	0.00	0.00	0.00	0.00	0.00	0.00	0.00	0.00	0.00	0.00	0.00
F2-SZ01- II	0.10	0.36	35.76	0.88	0.02	1.88	5.72	0.14	14.30	0.28	0.23	22.73	0.58	0.11	11.12	1.46	0.19	19.10	44.30	0.19	18.75	0.39	0.06	6.49
F3-SZ01- II	0.08	0.28	28.03	4.13	0.08	8.32	5.23	0.15	15.28	0.05	0.04	4.13	0.29	0.06	6.01	0.17	0.02	2.22	19.50	0.09	9.15	1.10	0.19	18.95
F4-SZ01- II	0.00	0.00	0.29	0.06	0.00	0.19	0.08	0.00	0.19	0.04	0.03	3.16	0.03	0.00	0.49	0.47	0.05	5.19	0.37	0.00	0.16	0.00	0.00	0.03
F1-SZ02-I	0.00	0.00	0.03	0.01	0.00	0.01	0.00	0.00	0.00	0.59	0.38	38.24	0.00	0.00	0.00	0.00	0.00	0.00	0.00	0.00	0.00	0.00	0.00	0.00
F2-SZ02-I	0.06	0.20	20.07	0.75	0.02	1.60	5.76	0.14	14.40	0.28	0.22	22.49	0.39	0.08	7.57	1.34	0.18	17.53	36.70	0.16	15.53	0.52	0.08	8.49
F3-SZ02-I	0.08	0.26	25.57	4.01	0.08	8.08	5.68	0.17	16.60	0.04	0.04	3.65	0.22	0.05	4.64	0.21	0.03	2.73	20.30	0.10	9.53	1.15	0.20	19.76
F4-SZ02-I	0.00	0.01	1.37	0.06	0.00	0.19	0.82	0.02	2.09	0.05	0.04	3.67	0.03	0.00	0.49	0.11	0.01	1.17	1.87	0.01	0.80	0.00	0.00	0.07
F1-SZ02-II	0.00	0.00	0.02	0.01	0.00	0.01	0.00	0.00	0.00	0.59	0.38	38.37	0.00	0.00	0.00	0.00	0.00	0.00	0.00	0.00	0.00	0.00	0.00	0.00
F2-SZ02-II	0.06	0.20	19.72	0.67	0.01	1.43	5.31	0.13	13.28	0.26	0.21	20.63	0.35	0.07	6.66	1.36	0.18	17.79	34.20	0.14	14.47	0.46	0.08	7.58
F3-SZ02-II	0.07	0.25	24.93	3.01	0.06	6.06	4.90	0.14	14.32	0.03	0.03	3.05	0.21	0.04	4.37	0.19	0.03	2.52	18.00	0.08	8.45	0.97	0.17	16.78
F4-SZ02-II	0.01	0.02	2.44	0.06	0.00	0.19	0.34	0.01	0.87	0.03	0.02	2.36	0.03	0.00	0.49	0.27	0.03	2.92	1.76	0.01	0.75	0.00	0.00	0.03
F1-SZ03-I	0.00	0.00	0.02	0.01	0.00	0.01	0.00	0.00	0.00	0.43	0.28	28.24	0.00	0.00	0.00	0.00	0.00	0.00	0.00	0.00	0.00	0.00	0.00	0.00
F2-SZ03-I	0.03	0.09	9.48	1.75	0.04	3.74	2.06	0.05	5.15	0.02	0.02	1.65	0.09	0.02	1.81	0.09	0.01	1.14	7.70	0.03	3.26	0.49	0.08	8.10
F3-SZ03-I	0.05	0.18	18.00	3.80	0.08	7.65	4.14	0.12	12.10	0.03	0.03	2.60	0.17	0.04	3.57	0.12	0.02	1.56	14.10	0.07	6.62	0.95	0.16	16.33
F4-SZ03-I	0.00	0.00	0.29	0.06	0.00	0.19	0.23	0.01	0.59	0.02	0.01	1.36	0.03	0.00	0.49	0.06	0.01	0.64	0.48	0.00	0.20	0.00	0.00	0.05
F1-SZ03-II	0.00	0.00	0.02	0.01	0.00	0.01	0.00	0.00	0.00	0.34	0.22	22.29	0.00	0.00	0.00	0.00	0.00	0.00	0.00	0.00	0.00	0.00	0.00	0.00
F2-SZ03-II	0.06	0.19	19.31	3.64	0.08	7.78	4.17	0.10	10.43	0.03	0.02	2.38	0.17	0.03	3.30	0.12	0.02	1.50	14.60	0.06	6.18	0.91	0.15	14.97
F3-SZ03-II	0.01	0.03	3.02	0.35	0.01	0.71	0.78	0.02	2.27	0.01	0.01	1.03	0.06	0.01	1.34	0.04	0.01	0.53	2.87	0.01	1.35	0.10	0.02	1.70
F4-SZ03-II	0.00	0.00	0.29	0.06	0.00	0.19	0.14	0.00	0.37	0.01	0.01	0.78	0.03	0.00	0.49	0.05	0.01	0.58	0.30	0.00	0.13	0.00	0.00	0.05
F1-SZ04-I	0.00	0.00	0.02	0.00	0.00	0.00	0.00	0.00	0.01	0.59	0.39	38.56	0.00	0.00	0.00	0.00	0.00	0.00	0.00	0.00	0.00	0.00	0.00	0.00
F2-SZ04-I	0.06	0.19	19.44	0.76	0.02	1.63	6.91	0.17	17.28	0.21	0.17	16.91	0.65	0.12	12.48	1.62	0.21	21.19	50.90	0.22	21.54	0.60	0.10	9.89
F3-SZ04-I	0.05	0.16	16.23	1.83	0.04	3.69	4.29	0.13	12.54	0.05	0.05	4.73	0.23	0.05	4.92	0.17	0.02	2.18	18.30	0.09	8.59	1.14	0.20	19.64
F4-SZ04-I	0.01	0.01	1.49	0.06	0.00	0.19	0.71	0.02	1.80	0.05	0.04	3.58	0.03	0.00	0.49	0.17	0.02	1.82	2.06	0.01	0.88	0.01	0.00	0.10
F1-SZ04-II	0.00	0.00	0.02	0.00	0.00	0.00	0.00	0.00	0.01	0.50	0.32	32.48	0.00	0.00	0.01	0.00	0.00	0.00	0.00	0.00	0.00	0.00	0.00	0.00
F2-SZ04-II	0.06	0.21	20.59	0.71	0.02	1.51	6.65	0.17	16.63	0.21	0.17	17.07	0.62	0.12	11.85	1.65	0.22	21.59	48.30	0.20	20.44	0.54	0.09	8.90
F3-SZ04-II	0.05	0.17	17.40	1.86	0.04	3.75	4.24	0.12	12.39	0.04	0.04	4.09	0.21	0.04	4.46	0.16	0.02	2.12	16.20	0.08	7.61	1.10	0.19	18.91
F4-SZ04-II	0.01	0.02	1.96	0.06	0.00	0.19	0.87	0.02	2.22	0.05	0.04	3.53	0.03	0.00	0.49	0.21	0.02	2.25	2.42	0.01	1.03	0.01	0.00	0.13

*F1*–*F4* fraction, *SZ01*–*SZ04* sample names, *I*, *II* series, *R* leachability, *R*% share of element in the specific fraction in percent

### Analytical methods

The chemical composition of ISSA was obtained using inductively coupled plasma mass spectrometry (ICP-MS) and inductively coupled plasma atomic emission spectroscopy (ICP-AES), performed by Bureau Veritas Minerals (formerly AcmeLabs Analytical Laboratories) in Vancouver, Canada. To determine the chemical composition of leachates, ICP-MS analyses were performed by Activation Laboratories Ltd. (Actlabs) in Ancaster, Canada.

X-ray diffraction (XRD) was used for mineralogical characterization ISSA samples. A Philips X’Pert (APD type) diffractometer with a PW 3020 vertical goniometer equipped with a curved graphite crystal monochromator (CuKα radiation, analytical range 2–64° 2Θ, step 0.02°, counting time 2 s/step) was used. For the phase identification Philips X’Pert software (associated with the ICDD database) was used. The analyses were performed at the Institute of Geological Sciences, Jagiellonian University in Krakow, Poland.

A field emission scanning electron microscope (SEM) Hitachi S-4700 combined with a Noran energy dispersive spectrometer was used for detailed microscopic observation of post-extraction solid residues. The residue grains mounted on carbon discs were coated with carbon. Observations were done at accelerating voltage of 20 kV and beam current of 10 mA, using the secondary electron (SE) imaging mode. The analyses were performed at the Institute of Geological Sciences, Jagiellonian University in Krakow, Poland.

## Results and discussion

### Chemical composition of ISSA

The studied ISSA was Si–Fe–P–Ca–Al dominated material, with relatively low content of heavy metals and potentially toxic elements and REE which enable the consideration of this material as solid mineral fertilizers (Kasina et al. 2019). The concentrations of major and minor elements are listed in Table 4.

**Table 4:** The bulk chemistry of studied samples based on ICP methods

		SZ01	SZ02	SZ03	SZ04
		Fly ash	Fly ash	Fly ash	Fly ash
Major elements					
Si	%	16.36	16.51	18.94	18.16
	mg/L	163,600	165,100	189,400	181,600
Al	%	4.17	4.36	3.92	4.53
	mg/L	41,700	43,600	39,200	45,300
Fe	%	11.08	9.97	8.71	10.26
	mg/L	110,800	99,700	87,100	102,600
Mg	%	2.09	2.21	2.28	2.26
	mg/L	20,900	22,100	22,800	22,600
Ca	%	9.03	9.62	8.85	7.09
	mg/L	90,300	96,200	88,500	70,900
Na	%	0.53	0.55	0.53	0.47
	mg/L	5300	5500	5300	4700
K	%	1.5	1.59	1.43	1.55
	mg/L	15,000	15,900	14,300	15,500
P	%	7.81	7.74	7.02	7.39
	mg/L	78,100	77,400	70,200	73,900
Mn	%	0.09	0.09	0.09	0.08
	mg/L	900	900	800	800
Minor elements					
As	mg/L	16.02	14.1	11.6	14.0
Cd	mg/L	7.0	4.8	5.0	5.7
Cr	mg/L	1046.52	779.76	827.64	526.68
Cu	mg/L	665.5	666.6	570.3	654.8
Mo	mg/L	25.5	20.6	18.3	21.5
Ni	mg/L	119.5	86.8	79.3	102.4
Pb	mg/L	138.0	127.4	125.9	151.6
Zn	mg/L	4472.0	3938.0	3550.0	3918.0
∑ REE	mg/L	90.86	101.37	96.81	106.38

### Characteristic of phosphorus rich mineral phases

The phosphorus present in the ISSA starting samples varied between 7 and 8%. These relatively high concentrations enable the comparison of ISSA to medium grade phosphorus ores (Kasina et al. 2020). The phosphorus in ISSA is usually present not as a free phosphate but combined aggregates or phases (Franz 2008); therefore, its identification is relevant. The results of XRD analyses indicated that apart from non-phosphorus mineral phases such as quartz, feldspar, and hematite, Fe–Mg–Ca phosphate of a whitlockite structure (22 ± 0.8%) and Fe–PO_4_ phase (PDF card 15–0655) (1.5 ± 0.3%) were present in the studied ashes (Kasina et al. 2019). The content of amorphous phase was estimated based on Rietveld refinement 19.9 ± 2.4 wt%.

**Fig. 2:**
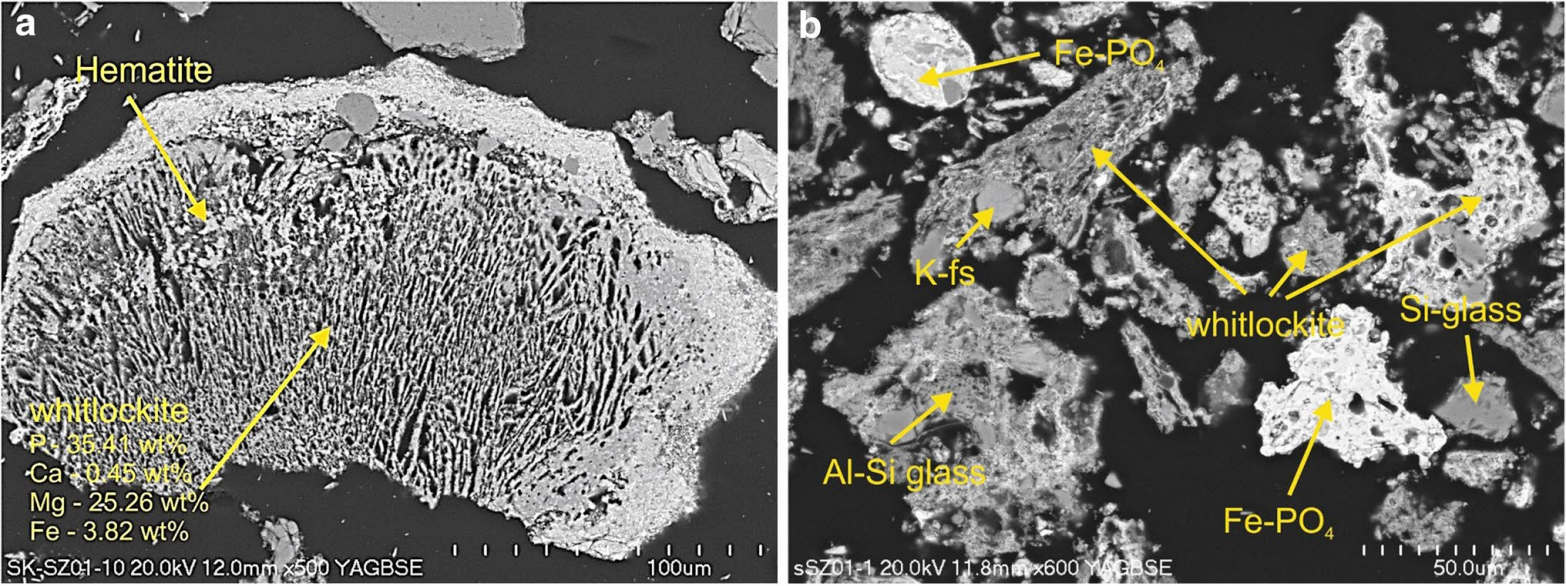
**a** Radially shaped whitlockite rich in Mg overgrowth with hematite. SEM BSE image mode. Magnification 500 × . **b** Fe–PO_4_ in the rounded form (left top side), and as irregular sinter (right, down side). SEM-BSE image mode, magnification 600 ×

The whitlockite-like minerals were characterized by various proportions in Fe, Mg, Ca, and P contents. They were radially shaped and sometimes overgrown with hematite (Fig. 2a, b). The Fe–PO_4_ were usually perfectly rounded (Fig. 2b). Also some amounts of phosphorus were detected in a matrix (~ 10 wt%), a shapeless assemblage composed of Si, Al, Fe, Ca, P, Mg, K, and Na in various proportions.

### Results of sequential extraction

The results of the sequential extraction showed that the highest amounts of phosphorus were released due to sulfuric acid leaching (on average 1470 mg L^−1^) causing the release of phosphorus combined with organic matter in F4 at the level of about 64%. The efficiency was, however, low, compared to other studies where 80–99% of phosphorus was released, e.g., Baldi et al. (2021); Biswas et al. (2009); Cohen (2009); Franz (2008); Wang et al. (2018). In addition, Fang et al. (2018) noted that sulfuric acid is the most efficient acid in phosphorus recovery due to higher concentration of hydrogen ions.

Sun et al. (2018) found out that in acid leaching with H_2_SO_4_, calcium, magnesium, potassium, and phosphorus are removed, thus we may assume that this reaction is responsible mostly for whitlockite dissolution, which is the most abundant phosphorus mineral phase in the studied ISSA. A dissolution of heavy metals and potentially toxic elements occurred, therefore purification process needs to be applied. The acid extract can be subjected to organic solvent treatment, as suggested by Baldi et al. (2021) or purified using chelating solutions, as suggested by Fang et al. (2018).

Precipitation of phosphates from the leachate may give additional information on the possible utilization of the leachates. As suggested by Liang et al. (2019), it was possible to synthetize high purity struvite from H_2_SO_4_ leachate, which can be used directly as fertilizer (Talboys et al. 2016; Nongqwenga et al. 2017).

The chelating compound indicated lower ability to extract phosphorus. The Na-EDTA leachant released on average 890 mg L^−1^ which was on average about 35%. The EDTA enables the formation of complexes with Fe^3+^ ions, thus stabilizing them in the solution, which prevents their co-precipitation with phosphorus (Anawati and Azimi 2020). Here, in the leachates of fraction F2 the highest concentrations of trace elements were detected. Among which around 50% belonged to the As; additionally 40% of Zn and over 20% of Mo were released. Also, significant concentrations of Cu and Cd, Ni, and Pb (over a dozen%) were measured. For this reason, Fang et al. (2018), recognized EDTA as a relevant pre-treatment agent for the total trace elements reduction in ISSA, which reduces contamination during subsequent phosphorus extraction with H_2_SO_4_ and additionally causes insignificant changes in the ash structure.

Only single percent of phosphorus was released in the reaction with deionized water and after leaching with NaOH. Also, a relatively low leaching rate of minor elements (REE and heavy metals and potentially toxic elements) occurred (Table 3). That might be due to the fact that metals were partially immobilized in insoluble phases and ash glassy matrix or leached out in the previous stages of extraction.

The sequential extraction led to the recovery of phosphorus of around 40–60% depending on the initial sample (Table 2), while as suggested by Cornel and Schaum (2009) phosphorus recovery rates can reach up to 90%.

Ruan et al. (2019) and Anawati and Azimi (2020) indicated that Al_2_O_3_ + Fe_2_O_3_ > 3 wt% and presence of impurities such as chlorine, fluorine, strontium, and heavy metals can affect P_2_O_5_ recovery and unwanted crystallization of Al and Fe salts. These salts are characterized by low solubility and low plant availability (Kalmykova and Karlfeldt Fedje, 2013). In the studied samples, the Al_2_O_3_ and Fe_2_O_3_ contents were higher than 20% (Table 4). Additionally, the high pH and the high Ca/P ratio may negatively influence the precipitation of phosphate minerals over calcium carbonates (Song et al. 2002). In the studied samples, the pH of the starting solution was over 9; the Ca/P ration oscillated around 1; and calcium carbonates were not detected. However, Stendahl and Jäfverström (2004) indicated that high content of Ca limits the phosphorus release. In that study, 90% of phosphorus was leached from the sewage sludge ash containing 3% of Ca, whereas only 65% of phosphorus was released from the sewage sludge ash containing 8% of Ca. In the studied samples, the Ca content was on average 8.5% (Table 2) what could strongly influence efficiency. Here, we may assume that the bulk chemical composition of the sample is relevant in phosphorus recovery. In addition, mineral composition of ISSA and the phosphorus speciation are important, since the solubility of different mineral phases containing phosphorus depends on chemical reagents.

**Fig. 3:**
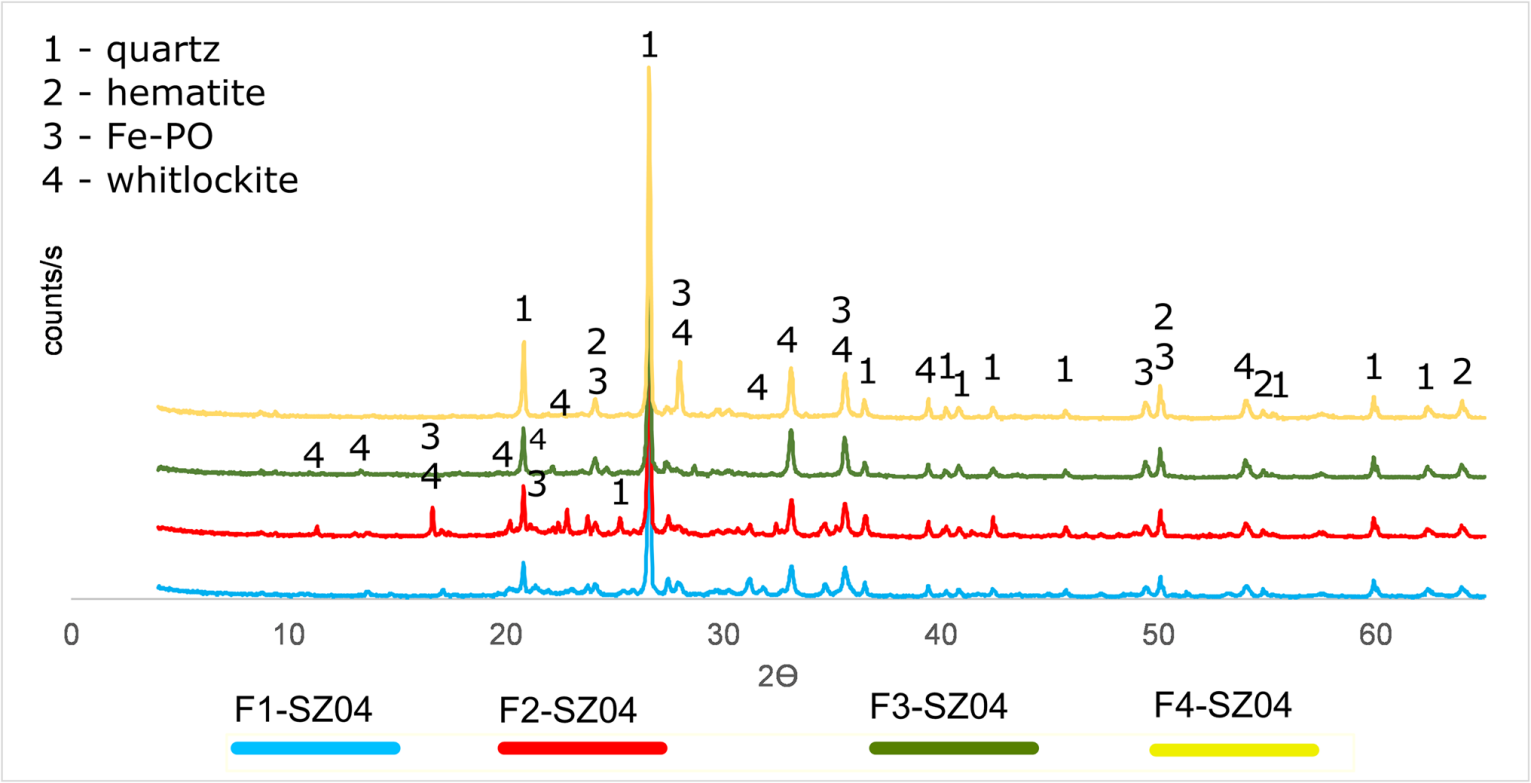
The XRD patterns based on sample SZ04 after each stage of sequential extraction

The results of X-ray diffraction can be an additional indicator for solubility of phosphorus phases and thus leaching efficiency. The XRD results indicated, however, similar mineral composition as in the starting ISSA samples (Fig. 3).

Only very slight variations in peak intensities were observed. That may be related to the fact that the sequential extraction efficiency was not high enough to cause dissolution and/or recrystallization of other mineral phases due to sequential extraction. The phosphorus cold have been immobilized as a result of closing it within matrix, which did not dissolve under the influence of leaching solutions.

Here, the conclusion appears that either the procedure used for sequential extraction requires repetition, or another more efficient procedure should be applied to obtain more satisfactory results.

### Results of sequential extraction — the influence of the results on possible phosphorus bioavailability

The recovery of phosphorus from ISSA is an important issue in anthropogenic phosphorus cycle and sustainable economy; however, we must keep in mind that not all recovered phosphorus is mobile and bioavailable; therefore, it may negatively influence environment by eutrophication. It is, thus, important to evaluate the bioavailability of phosphorus recovered from waste and its possible usage in the fertilizer production. The studied ISSA fulfilled requirements for non-hazardous waste (Kasina et al. 2021) and usage as solid mineral fertilizers are fulfilled following national and EU norms (Kasina et al. 2020) due to high content of macro-nutrients (K_2_O + P_2_O_5_) and acceptable content of potentially toxic elements (As, Pb, Cd, Hg).

In the used extraction procedure, only phosphorus associated with carbonates and the one associated with Fe and Al oxides can be considered mobile and bioavailable, so F1, F2, and F4, respectively, whereas F3 is considered non-bioavailable (Wang et al. 2013). The phosphorus compounds released as a result of the reaction with H_2_SO_4_ show a significant potential for recovery; however, the release of heavy metals and potentially toxic elements occur and, this fraction indicates very limited bioavailability.

## Conclusions

The obtained results highlight the added value of the potential use of ISSA when considering optimization of P recovery from ISSA by wet chemical methods.

A four-stage sequential extraction based on the modified Golterman method is efficient enough in recovery of phosphorus.

Phosphorus minerals are still present in post extraction solid samples, indicating not complete dissolution of phosphorus-rich mineral phases.

Acid leaching caused not only higher phosphorus extraction than leaching using basic and chelating solutions, but also higher extraction of heavy metals and potentially toxic elements.

Even though the overall extraction level was sufficient, it is assumed that better result might be achieved by multiplication of procedure.

## Data Availability

All the data generated or analyzed during this study are included in this published article.

## References

[CR1] Anawati J, Azimi G (2020). Recovery and separation of phosphorus as dicalcium phosphate dihydrate for fertilizer and livestock feed additive production from a low-grade phosphate ore. RSC Adv.

[CR2] Antonkiewicz J (2010). Effect of sewage sludge and furnace waste on the content of selected elements in the sward of legume-grass mixture. J Elem.

[CR3] Antonkiewicz J, Kołodziej B, Bielińska EJ, Gleń-Karolczyk K (2018). The use of macroelements from municipal sewage sludge by the multiflora rose and the Virginia fanpetals. J Ecol Eng.

[CR4] Alewell C, Ringeval B, Ballabio CA, Robinson DA, Panagos P, Borrelli P (2020) Global phosphorus shortage will be aggravated by soil erosion. Nat Commun 11, 4546 (2020). 10.1038/s41467-020-18326-7 10.1038/s41467-020-18326-7 PMC7486398 32917863

[CR5] Baldi M, Martinotti A, Sorlini S, Katsoyiannis IA, Abbà A, CarnevaleMiino M, Collivignarelli MC (2021). Extraction and purification of phosphorus from the ashes of incinerated biological sewage sludge. Water.

[CR6] Belhaj D, Elloumi N, Jerbi B, Zouari M, Abdallah FB, Ayadi H, Kallel M (2016). Effects of sewage sludge fertilizer on heavy metal accumulation and consequent responses of sunflower (Helianthus annuus). Environ Sci Pollut Res.

[CR7] Biswas BK, Inoue K, Harada H, Ohto K, Kawakita H (2009). Leaching of phosphorus from incinerated sewage sludge ash by means of acid extraction followed by adsorption on orange waste gel. J Environ Sci.

[CR8] Cohen Y (2009). Phosphorus dissolution from ash of incinerated sewage sludge and animal carcasses using sulphuric acid. Environ Technol.

[CR9] Commission Decision of 18 December 2014 amending Decision 2000/532/EC on the list of waste pursuant to Directive 2008/98/EC of the European Parliament and of the Council (n. d.)

[CR10] Cordell D, Drangert J-O, White S (2009). The story of phosphorus: global food security and food for thought. Glob Environ Chang.

[CR11] Cornel P, Schaum C (2009). Phosphorus recovery from wastewater: needs, technologies and costs. Water Sci Technol.

[CR12] Desmidt E, Ghyselbrecht K, Zhang Y, Pinoy L, Van der Bruggen B, Verstraete W, Rabaey K, Meesschaert B (2015). Global phosphorus scarcity and full-scale P-Recovery techniques: a review. Crit Rev Environ Sci Technol.

[CR13] European Commission. Report on Critical Raw Materials for the EU (2014) In Report of the Ad-Hoc Working Group on defining critical raw materials; Raw Materials Supply Group: Brussels, Belgium, 2014.

[CR14] Fang L, Li JS, Guo MZ, Cheeseman CR, Tsang DCW, Donatello S, Poon CS (2018). Phosphorus recovery and leaching of trace elements from incinerated sewage sludge ash (ISSA). Chemosphere.

[CR15] Franz M (2008). Phosphate fertilizer from sewage sludge ash (SSA). Waste Manage.

[CR16] Golterman HL (1996). Fractionation of sediment phosphate with chelating compounds: a simplification, and comparison with other methods. Hydrobiologia.

[CR17] Gorazda K, Wzorek Z, Tarko B, Nowak AK, Kulczycka J, Henclik A (2013). Phosphorus cycle - possibilities for its rebuilding. Acta Biochim Pol.

[CR18] Guidance on classification of waste according to EWC-Stat categories (2010) Supplement to the Manual for the Implementation of the Regulation (EC) No 2150/2002 on Waste Statistics. EUROSTAT

[CR19] Jaffer Y, Clark T, Pearce P, Parsons S (2002). Potential phosphorus recovery by struvite formation. Water Res.

[CR20] Kalmykova Y, Karlfeldt Fedje K (2013). Phosphorus recovery from municipal solid waste incineration fly ash. Waste Manage.

[CR21] Kasina M, Kajdas B, Michalik M (2021) The leaching potential of sewage sludge and municipal waste incineration ashes in terms of landfill safety and potential reuse. Sci Total Environ 791. 10.1016/j.scitotenv.2021.148313 10.1016/j.scitotenv.2021.148313 34139499

[CR22] Kasina M, Kowalski P, Kajdas B, Michalik M (2020). Assessment of valuable and critical elements recovery potential in ashes from processes of solid municipal waste and sewage sludge thermal treatment. Resources.

[CR23] Kasina M, Wendorff-Belon M, Kowalski PR, Michalik M (2019). Characterization of incineration residues from wastewater treatment plant in Polish city: a future waste based source of valuable elements?. J Mater Cycles Waste Manage.

[CR24] Lamastra L, Suciu NA, Trevisan M (2018) Sewage sludge for sustainable agriculture: contaminants’ contents and potential use as fertilizer. Chem Biol Technol Agric 5(1). 10.1186/s40538-018-0122-3

[CR25] Le Corre KS, Valsami-Jones E, Hobbs P, Parsons SA (2009). Phosphorus recovery from wastewater by struvite crystallization: a review. Crit Rev Environ Sci Technol.

[CR26] Liang S, Chen H, Zeng X, Li Z, Yu W, Xiao K, … Yang J (2019) A comparison between sulfuric acid and oxalic acid leaching with subsequent purification and precipitation for phosphorus recovery from sewage sludge incineration ash. Water Res. 10.1016/j.watres.2019.05.022 10.1016/j.watres.2019.05.022 31100578

[CR27] Ruan Y, He D, Chi R (2019). Review on beneficiation techniques and reagents used for phosphate ores. Minerals.

[CR28] Meng X, Chen WW, Wang YY, Huang ZR, Ye X, Chen LS, Yang LT (2021) Effects of phosphorus deficiency on the absorption of mineral nutrients, photosynthetic system performance and antioxidant metabolism in Citrus grandis. PloS One 16(2). 10.1371/journal.pone.0246944 10.1371/journal.pone.0246944 PMC7888624 33596244

[CR29] Nongqwenga N, Muchaonyerwa P, Hughes J, Odindo A, Bame I (2017). Possible use of struvite as an alternative phosphate fertilizer. J Soil Sci Plant Nutr.

[CR30] Schroder JJ, Cordell D, Smit AL, Rosemarin A (2009) Sustainable use of phosphorus, European Union tender project ENV.B.1/ETU/2009/0025). Report 357, Plant Research International, Wageningen University and Research Centre. 122 pp. Wageningen, The Netherlands

[CR31] Shu L, Schneider P, Jegatheesan V, Johnson J (2006). An economic evaluation of phosphorus recovery as struvite from digester supernatant. Biores Technol.

[CR32] Song Y, Hahn HH, Hoffmann E (2002) The effect of carbonate on the precipitation of calcium phosphate. Environ Technol 23(2):207–215. 10.1080/09593332508618427 10.1080/09593332508618427 11950073

[CR33] Steffen W, Richardson K, Rockström J, Cornell SE, Fetzer I, Bennett EM (2015). Planetary boundaries: guiding human development on a changing planet. Science.

[CR34] Stendahl K, Jäfverström S (2004). Recycling of sludge with the Aqua Reci process. Water Sci Technol.

[CR35] Sun J, Xiu Y-F, Huang K, Yu J-T, Alam S, Zhu H-M, Guo Z-C (2018). Selective recovery of phosphorus from acid leach liquor of iron ore by garlic peel adsorbent. RSC Adv.

[CR36] Talboys PJ, Heppell J, Roose T, Healey JR, Jones DL, Withers PJA (2016). Struvite: a slow-release fertiliser for sustainable phosphorus management?. Plant Soil.

[CR37] Van Dijk KC, Lesschen JP, Oenema O (2016). Phosphorus flows and balances of the European Union Member States. Sci Total Environ.

[CR38] Wang Q, Li J-S, Tang P, Fang L, Poon CS (2018). Sustainable reclamation of phosphorus from incinerated sewage sludge ash as value-added struvite by chemical extraction, purification and crystallization. J Clean Prod.

[CR39] Wang C, Zhang Y, Li H, Morrison RJ (2013). Sequential extraction procedures for the determination of phosphorus forms in sediment. Limnology.

[CR40] Yang F, Chen J, Yang M, Wang X, Sun Y, Xu Y, Qian G (2019). Phosphorus recovery from sewage sludge via incineration with chlorine-based additives. Waste Manage.

